# Patterns and roles of lignan and terpenoid accumulation in the reaction zone compartmentalizing pathogen-infected heartwood of Norway spruce

**DOI:** 10.1007/s00425-022-03842-1

**Published:** 2022-02-10

**Authors:** Nina Elisabeth Nagy, Hans Ragnar Norli, Monica Fongen, Runa Berg Østby, Inger M. Heldal, Jahn Davik, Ari M. Hietala

**Affiliations:** 1 Norwegian Institute of Bioeconomy Research, P.B. 115, 1431 Ås, Norway; 2 Norwegian Institute of Bioeconomy Research, P.B. 2609, 7734 Steinkjer, Norway; 3 Faculty of Health, Welfare and Organisation, Østfold University College, P.B. 700, 1757 Halden, Norway

**Keywords:** GC–MS, Host–pathogen interaction, Parenchyma, Traumatic resin ducts, White rot

## Abstract

**Main conclusion:**

Lignan impregnation of the reaction zone wood protects against oxidative degradation by fungi. Traumatic resin canals may play roles in the underlying signal transduction, synthesis, and translocation of defense compounds.

**Abstract:**

Tree defense against xylem pathogens involves both constitutive and induced phenylpropanoids and terpenoids. The induced defenses include compartmentalization of compromised wood with a reaction zone (RZ) characterized by polyphenol deposition, whereas the role of terpenoids has remained poorly understood. To further elucidate the tree–pathogen interaction, we profiled spatial patterns in lignan (low-molecular-weight polyphenols) and terpenoid content in Norway spruce (*Picea abies*) trees showing heartwood colonization by the pathogenic white-rot fungus *Heterobasidion parviporum*. There was pronounced variation in the amount and composition of lignans between different xylem tissue zones of diseased and healthy trees. Intact RZ at basal stem regions, where colonization is the oldest, showed the highest level and diversity of these compounds. The antioxidant properties of lignans obviously hinder oxidative degradation of wood: RZ with lignans removed by extraction showed significantly higher mass loss than unextracted RZ when subjected to Fenton degradation. The reduced diversity and amount of lignans in pathogen-compromised RZ and decaying heartwood in comparison to intact RZ and healthy heartwood suggest that α-conindendrin isomer is an intermediate metabolite in lignan decomposition by *H. parviporum*. Diterpenes and diterpene alcohols constituted above 90% of the terpenes detected in sapwood of healthy and diseased trees. A significant finding was that traumatic resin canals, predominated by monoterpenes, were commonly associated with RZ. The findings clarify the roles and fate of lignan during wood decay and raise questions about the potential roles of terpenoids in signal transduction, synthesis, and translocation of defense compounds upon wood compartmentalization against decay fungi.

**Supplementary Information:**

The online version contains supplementary material available at 10.1007/s00425-022-03842-1.

## Introduction

When considering the longevity, large size, and ecological dominance of trees, one can conclude that these plants have developed highly efficient defense mechanisms against abiotic and biotic stressors. The ultimate purpose of tree stem defenses is to safeguard the translocation of photosynthates in the inner bark, the transpiration stream in sapwood (outer part of the stem xylem where sap flows), and protect the meristematic tissue residing between phloem and sapwood. The vast amounts of carbohydrates present in the different tissues of trees attract a wide range of evolutionary diverse pests, which necessitate a broad spectrum of specific defense mechanisms. Most of the biomass of trees resides in tree stems, which are protected by constitutive structural and chemical barriers. These barriers are coupled with inducible responses, should they become compromised (Franceschi et al. 2005; Morris et al. 2020).

The bulk of tree xylem is formed by tracheary elements that undergo programmed cell death upon maturation and thus lack any inducible defense responses. The inducible responses are mounted by xylem parenchyma, whose amount and pattern of organization vary between tree species. Along with increase in tree stem diameter, the older central part of stem xylem is transformed into heartwood. This process involves the transformation of parenchyma energy reserves into extractives and subsequent death of the parenchyma cells in the heartwood (Hillis 1968; Bamber 1976).

Trees have generally both radially and axially orientated parenchyma in sapwood xylem (Morris et al. 2016). In conifers, the living parenchyma are primarily associated with the rays, initiating in phloem and extending radially across the xylem, and with the radially and axially orientated resin channels. This interconnected three-dimensional network provides a pathway for defense signaling, translocation, and biosynthesis of defense-related compounds in tree sapwood (Nagy et al. 2000; Morris et al. 2016). The extent of the parenchyma cell network and the nature of the induced defense compounds differ considerably between tree species (Morris et al. 2020), presumably because of adaptation to different biotic and abiotic selection pressures.

Durability of heartwood toward wood decay is largely defined by the amount and nature of stored extractives. For example, species of fir (*Abies* spp.) and spruce (*Picea* spp.) have generally a low extractive content in heartwood (Willför et al. 2004a, b, c), which makes them more susceptible to wood decay pathogens than extractive-rich species such as trees in the genus *Pinus*. Consequently, by the time of final harvest, commonly 20% or more of the fir and spruce trees show heartwood decay by pathogenic white-rot fungi of the genus *Heterobasidion* (Puddu et al. 2003; Hietala et al. 2016). As entrance and transfer points, these fungi utilize root and stem wounds and root connections formed between neighboring trees. Once established in heartwood, the infection challenges sapwood defense responses to expand the decay column laterally.

**Fig. 1:**
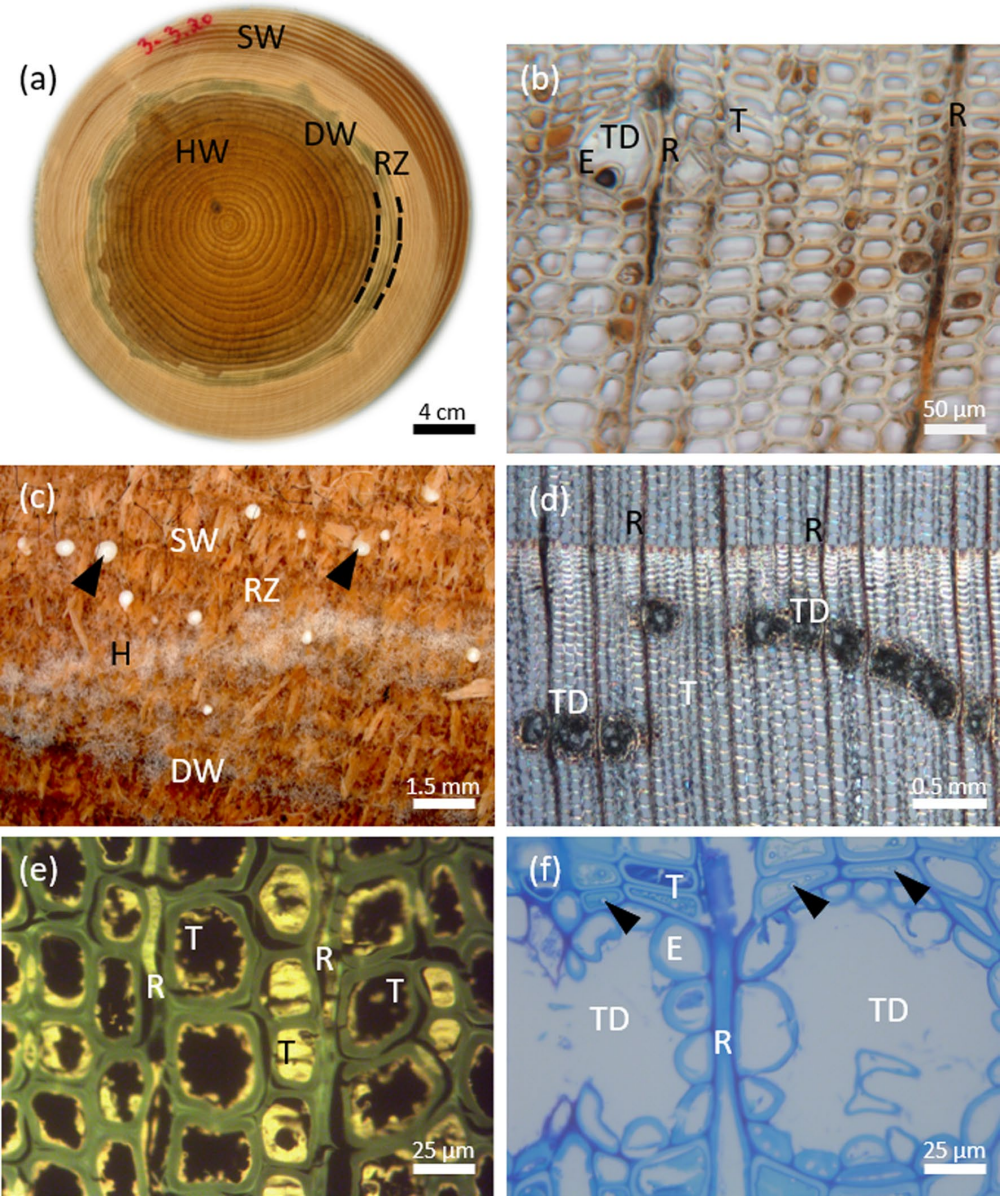
Morphological characteristics of wood decayed by the fungus *H. parviporum,* shown in transverse sections from the stem of Norway spruce: **a** Stem disc taken at 3.2 m height and showing sapwood (SW), the reaction zone (RZ), the discolored heartwood (DW) that coincides with the fungal colonization frontier, and heartwood (HW). Note that the stem disc has been exposed to air and subsequent oxidation leading to changes in coloration. **b** Micrograph of wood cells from the reaction zone with high content of dark browned oxidized fillings of polyphenolics in tracheids (T), ray parenchyma (R), and epithelial cells surrounding the traumatic resin duct (TD). **c** Section of the wood disc showing the reaction zone (RZ) and discolored tissue (DW) with conidiophores of *H. parviporum* forming a whitish mat of aerial hyphae (H), and resin droplets (arrow heads) exuded from the resin canals beneath. **d** Traumatic resin ducts (TD) in continuous rows and associated with rays (R) and tracheids (T). **e** Autofluorescence of polyphenolics in tracheids (T) and rays (R). **f** Thin section of the traumatic resin ducts (TD) with epithelial cells (E) lining the ducts, associated with radial ray parenchyma (R) and cells with phenol accumulation (turquoise color, the tissue sections (1.2 µm) were stained with Stevenel’s Blue (del Cerro et al. 1980); some enclosing fungal hyphae (arrow heads)

The defense response, forming at the interface between sapwood and pathogen-colonized heartwood, is referred to as a reaction zone (Fig. 1). This zone can be considered as pathological heartwood as it arises via sacrifice of inner sapwood in response to heartwood colonization by the pathogen. This compartmentalization process resists further loss of functional sapwood and spread of invading organisms and the associated damage (Shigo 1984). When reaction zone boundaries fail, a volume of wood is colonized with little or no expression of characteristic reaction zone responses, until ultimately a new reaction-zone boundary is established (Pearce 1991).

In Norway spruce (*Picea abies*), the reaction zone responses involve impregnation of wood by a large group of polyphenolic compounds belonging to lignans, coupled with elevated pH and cation accumulation in the tissue (Shain 1971; Hammerbacher et al. 2020). Besides polyphenols, the inducible defense compounds also include terpenoids, a main compound group in conifer resin and considered important as antifeedants against bark and foliage attacking insects of conifers (Franceschi et al. 2005). Bark inoculation of Norway spruce by *Heterobasidion* species triggers the formation of traumatic resin channels (Krekling et al. 2004), and certain terpenes have shown antifungal activities toward *Heterobasidion* species under in vitro conditions (Kusumoto et al. 2014). However, the induction and potential defense roles of terpenes when Norway spruce sapwood is challenged by heartwood colonization remain unknown.

As described above, the reaction zone at the sapwood–heartwood interface appears to form a barrier for horizontal spread of pathogenic fungi into the sapwood. The main objective of the present study was to increase the understanding of host–pathogen interaction by profiling the diversity and level of defense compounds in intact and pathogen-colonized xylem tissues. We hypothesize that defense of Norway spruce trees toward *Heterobasidion* wood decay fungi involves products of biosynthetic pathways of both lignans and terpenoids, and that pathogen colonization of wood influences the amount and composition of the associated defense compounds. These hypotheses were pursued by a detailed vertical and horizontal profiling of the distribution pattern of lignans and terpenes in stems of diseased and healthy trees.

## Materials and methods

### Collection and storage of materials

Samples were collected upon clear-cutting of a Norway spruce stand at Lillenga, Nesodden (59°44ʹ48ʹʹN 10°36ʹ10ʹʹ), done by a forest harvester on June 7th, 2016. The average length of the trunks was 25 m, and the average stem diameter at stump level was 39.7 cm. The material studied for the infected trees, referred to in the text as ‘diseased trees’, included freshly cut, 3.2-m-long bolts, from stem base of three randomly chosen trees (trees #1, 2, 3) showing typical signs of *Heterobasidion* caused heartwood decay originating from roots (i.e., decay columns occupied the entire heartwood) and formation of a reaction zone around the entire sapwood/heartwood interface. Basal logs from three randomly chosen trees without any signs of decay (trees #4, 5, 6), referred to in the text as ‘healthy trees’, were taken to serve as reference. Stem slices (5–10 cm thick) were cut with a chainsaw from the base (referred to as 0 m or stump level in the text), 1.6 and 3.20 m heights of the log for each diseased tree and from the base, and 3.20 m height from the healthy trees. The slices were stored at -20 ºC overnight. An outline of the experimental set up and sampling design is given in Table 1.

**Table 1:** Sampling design and total number of samples, as well as subsamples, collected from different tissue zones of stems of diseased (*n* = 3) and healthy Norway spruce trees (*n* = 3) at different heights

Status	Trees	Height (m)	Tissue zones with [subzones]								
			HW	DW	RZ^a^	[i	m	o]	SW^a^	[i	o]
Diseased	3	0.0	2	3	8	[3	2	3]	6	[3	3]
		1.6	3	3	7	[3	1	3]	6	[3	3]
		3.2	3	3	5	[2	0	3]	6	[3	3]
Healthy	3	0.0	3	–	–				6	[3	3]
		1.6	0	–	–				0		
		3.2	3	–	–				6	[3	3]

Subsamples were collected only from sapwood and reaction zone (i = inner; m = middle; o = outer). Main tissue zones are abbreviated as follows: HW = heartwood; DW = discolored wood; RZ = reaction zone; SW = sapwood. Tissue zones not existing in healthy wood are delineated with a hyphen, -

^a^For statistical analyses, the subsamples were categorized to their main tissue zone

Additional discs, 0.5–1 cm in thickness, were cut from each tree and each sampling height by means of a bandsaw—after bark removal and cleaning with running tap water, the discs were imaged by a scanner. For verification of the area colonized by *Heterobasidion*, the discs were incubated in plastic bags kept in darkness at room temperature for 6 days, and the area hosting conidiophores characteristic to *Heterobasidion* species was determined by a binocular loupe. Samples were also taken from the colonized wood to isolate fungal DNA for identification of the *Heterobasidion* species.

### Identification of *Heterobasidion* species associated with the analyzed trees

Samples from heartwood, the adjacent discolored wood included, were pulverized in liquid N_2_-chilled Eppendorf tubes with a Retsch 300 mill (Retsch Gmbh, Haan, Germany). Up to 30 mg of tissue was processed with DNeasy Plant Mini Kit (Qiagen, Hilden, Germany) according to the manufacturer’s instructions, to a final elution volume of 50 μl. The real-time PCR assays for detection of *Heterobasidion annosum* and *H. parviporum*, the two *Heterobasidion* species present in Norway, were conducted in singleplex conditions in Takyon Low ROX Probe MasterMix dTTP Blue (Eurogentec, Seraing, Belgium), with primers and probes described by Ioos et al. (2019) and synthesized by Eurogentec.

The *H. annosum* qPCR assay included the forward primer 5′-CGTCGCCTTAATGATTTCATAAG-3′, the reverse primer 5′-TGTCACTGTACTGTTTCTTTAGC-3′ and the probe FAM-ACCATACAYGTTGGCGGGAACCTC-BHQ1. The *H. parviporum* qPCR assay included the forward primer 5′-CAATCGTATGGGGTCATTGTAA-3′, the reverse primer 5′-CACATCCGCCATGTCCC-3′ and the probe TAMRA-GATCTGCGAGCCCGACGAACCG-BHQ2. Real-time PCR amplifications were carried out in an Applied Biosystems ViiA 7 system (ThermoFisher, Waltham, MA, USA) with standard, instead of fast, cycling and the same PCR cycling parameters as described by Ioos et al. (2019). Two technical replicates were prepared for each sample. DNA obtained from mycelia of confirmed *H. annosum* and *H. parviporum* strains were used as references.

### Microscopy

For microscopy, a wood segment spanning from pith to living sapwood was removed from each of the frozen stem discs and 5-mm-wide wood blocks were cut out within the reaction zone and discolored wood (Fig. 1a). The specimens were fixed, embedded in L.R White resin (Electron Microscopy Sciences, Hatfield, PA, USA), and sectioned as previously described by Nagy et al. (2000). The tissue sections (1.2 µm) were stained with Stevenel’s Blue (del Cerro et al. 1980) for general observations. Ferric chloride was used to stain phenolic compounds (de Neergaard 1997), and unstained sections were used for detection of polyphenol auto-fluorescence (Franceschi et al. 1998). Thick sections (20 µm) from unprocessed wood blocks were cut on a vibratome for study of traumatic resin ducts. All images were taken with a Leica DMR light microscope at bright field and epifluorescence mode, respectively. Blue light (450–490 nm) and a long-band-pass filter (> 520 nm) was used for excitation and visualization of polyphenolics.

### Sampling and pretreatment for lignan and terpene analysis

The different zones present in stem slices were identified visually (Fig. 1a) and samples representing zones of interest were cut out. Wood delimited to sapwood and showing a yellow–brown–greenish color in freshly cut stem sections was considered as reaction zone, and the adjacent wood region with a violet–dark brown color in freshly cut stem sections was considered as discolored wood (Fig. 1a).

For the diseased trees, samples were taken from outer and inner sapwood, outer and inner reaction zone (in case the reaction zone was wide, also an additional sample was taken from the central part of this tissue), the discolored heartwood adjacent to reaction zone and outer heartwood (Table 1) and stored in small envelope paper bags at −20 °C. For the healthy trees, samples were taken from outer sapwood, inner sapwood, and outer heartwood (Table 1). The samples from each tissue were cut to smaller fragments, frozen in liquid N_2,_ milled in a Retsch MM 300 mill for 2 min at 30 rpm/s (resulting particle size 0.5–1 mm), lyophilized for 48 h in a freeze dryer, and thereafter stored at −20 °C until extraction.

Wood powder (200 mg) was placed on cellulose filters (10 × 50 mm, Munktell Filters, Grycksbo, Sweden), which were sealed with wads of cotton wool. Both the filters and the cotton wads were purified in n-hexane p.a. prior to use. For extraction of terpenes, *n*-hexane p.a. (25 ml) was added to the extraction bottles. The filters and the bottles were placed in a fluidized bed extraction (FBE) unit (FexIKA 50, IKA Werke GmbH & Co, Staufen, Germany), and the solvent was subjected to 30 cycles of heating and cooling (90 °C for 6 min, cooling to 50 °C). Extraction of lignans was performed with acetone p.a. (25 ml), 30 cycles of heating and cooling (78 °C for 6 min, cooling to 40 °C). The two extracts were stored in darkness, in sealed glass bottles at 4 °C.

### Analysis of lignans

To one milliliter of the acetone extract, 5 μg of nonadecanoic acid (Merck KGaA, Darmstadt, Germany, purity ≥ 98%) was added as an internal standard, and the sample was evaporated to complete dryness with nitrogen at 40 °C in a heating block. The lignans were derivatized to obtain trimethylsilyl (TMSi) ethers with free hydroxyl groups by adding 200 μl of N,O-bis(trimethylsilyl)trifluoroacetamide (BSTFA) containing 1% trimethylchlorosilane (TMCS) 99:1 v/v (Supelco, Bellefonte, PA, USA), followed by incubation at 70 °C for 30 min.

The measurements were carried out on a Hewlett Packard 6890 N gas chromatograph (GC) connected to a Hewlett Packard 5973 mass spectrometer (MS). The gas chromatograph was equipped with an Agilent 6890 autosampler and a Gerstel (Mühlheim Ruhr, Germany) programmable temperature vaporizing (PTV) injector with a multibaffle liner. The separation column was a fused silica Agilent HP-5MSUI 30 m with an internal diameter of 0.25 mm and 0.25 µm film thickness. The temperature program was as follows: 2 min at 65 °C, temperature increase of 6 °C min^−1^ to 300 °C with a hold for 10 min, total time 51.17 min. The PTV program was as follows: injection volume 5 µl, the solvent vent temperature was kept at 60 °C for 1.8 min with a solvent vent flow at 10.0 ml min^−1^. The split valve was closed after 1.9 min, and the injector temperature was raised (12 °C s^−1^) to 300 °C for 2 min. The mass spectrometer was operated in a scan mode from *m*/*z* 50 to 750 Da, threshold 50 and 1.1 scans/s.

For the identification of lignans, commercially available standards of α-conindendrin, secoisolariciresinol, pinoresinol, 7-hydroxy-matairesinol isomers, matairesinol, and lariciresinol supplied from Merck KGaA were used. The other lignans were identified by comparing mass spectra from literature (Ekman 1976; Yamamoto et al. 2004; Willför et al. 2005, 2006; Smeds et al. 2012). For screening and quantification of samples, Automatic Masspectra Deconvolution Identification Software (AMDIS version 2.62, NIST-08) was employed, with a custom-made database of the identified lignans linked to Kovàts retention indices (Kovàts 1958). The concentration estimates were calculated relative to the amount of internal standard and expressed as µg g^−1^ DW equivalent to nonadecanoic acid (Zhao et al. 2011; Flø et al. 2018).

### Analysis of terpenes

Five micrograms of the internal standard pentadecane (Merck KGaA, purity ≥ 99%) was added to 1.0 ml of hexane extract of each sample. Instrument settings, GC column type, temperature program, and MS parameters were as described by Dalen et al. (2015). For the verification of α-pinene, β-pinene, sabinene, verbenone, camphene, γ-terpinene, borneol, para-cymene, limonene, α-cedrene, α-cubebene, α-gurjunene, caryophyllene oxide, longycyclene, sativene, and α-terpineol, commercially available standards were supplied from Merck KGaA and Chiron AS (Trondheim, Norway). Germacrene-D was kindly supplied by Toby Bruce (Rothamstead, UK). The other terpenes were identified by use of NIST-08 mass spectra library.

For screening and quantification of terpenes, the same AMDIS software as employed for lignans was used with a database containing 1187 volatile compounds, these including the above-mentioned terpenes. Terpenes were quantified as pentadecane equivalents by dividing the peak areas of single terpenes from the total ion chromatogram by the peak area of the internal standard pentadecane as described by Dalen et al. (2015) and expressed as µg g^−1^ DW equivalent to pentadecane (Zhao et al. 2011; Flø et al. 2018).

For reference purpose, we also analyzed resin formed in axial traumatic canals associated with reaction zone in the three diseased trees, and resin associated with a resin pocket formed in the sapwood of the diseased tree #2 at 3.2 m height. Resin exuding from traumatic canals (Fig. 1c) at the surface of the wood discs taken at stump level of the diseased trees was picked up from approximately ten traumatic canals per each diseased tree with a tweezer. Resin was similarly collected from the resin pocket, and these samples were dissolved in 1.0 ml of hexane directly and subjected to the analytical pipelines described above.

### Fenton reaction induced wood mass loss

Durability of wood against oxidative degradation by decay fungi can be mimicked by measuring mass loss of wood subjected to Fenton reaction, a widely used degradation test for organic material, involving rapid generation of non-selective hydroxyl (OH) radicals that decompose organic molecules. To compare durability of reaction zone and sapwood, wood powder of these zones was subjected to degradation by Fenton reagents as described in Belt et al. (2017). Both unextracted and acetone extracted powders from reaction zone of diseased trees and inner sapwood of healthy trees were used as material to consider the extent to which wood durability can be assigned to extractives. Briefly, 100 mg of wood powder (1% wv^−1^) was incubated in 50 mM acetate buffer (pH 4.0) with 1 mM FeCl_2_ and 1.5% H_2_O_2_ for 72 h with constant shaking at 200 rpm at room temperature. Thereafter, the wood powder was filtrated through a preweighed whiteband filterpaper (Whatman 589/2 Ø 125 mm Ref. No. 10 300 111), washed with Elga-water, dried overnight at 105 °C, and weighed to determine mass loss. Five replicates were prepared for each sample.

### Statistical analyses

The distributions of the individual terpene and lignan contents were positively skewed; hence, the data were subjected to log transformation before further processing. The sub-tissue samples from sapwood (outer and inner) and reaction zone (outer, inner, and middle zones) were recorded as RZ and SW, respectively (Table 1). Mean values of single lignans and terpenes, total lignan, and total terpene contents were then obtained within health status, wood zone, and sampling height using the ‘aggregate’ function in R (R Core Team 2019). These mean values were standardized and subsequently used for exploratory data analysis by principal component analysis (PCA) and heatmaps combined with hierarchical clustering, using the ‘factoextra’ and ‘gplots’ packages (Kassambara and Mundt 2020; Warnes et al. 2020) in R. To consider status, sampling height, and tissue-type specific differences in the amount of lignans and terpenes, ANOVA was performed on log-transformed raw data using a nested linear mixed model (LMM) analyses according to the following model:

$$y_{ijkl} = \, \mu \, + \, S_{i} + \, H_{j} + \, Z_{k(i)} + \, tS_{li} + \, tHZ_{ljk} + \, \omega_{ijkl} ,$$

where *y*_*ijkl*_ is the log-transformed observation; µ is the overall mean; *S*_*i*_ is the fixed effect of the *i*th status of the tree (*i* = 1,2; healthy, diseased respectively); *H*_*j*_ is the fixed effect of the *j*th height above ground (*j* = 1,2,3; 0, 1.6, and 3.2 m respectively); *Z*_*k*(*i*)_ is the nested fixed effect of the *k*th tissue within status (S) from which the sample was taken (*k* = 1,..,4; heartwood, sapwood, discolored wood, reaction zone, respectively); *tS*_*li*_ is the nested random effect of the *l*th tree (*t*) (*l* = 1,2,3 and 4,5,6) within status (*S*) (*i* = 1,2), and *tHZ*_*ljk*_ is the nested random effect of the *k*th tissue zone (*Z*) within the *j*th height (*H*) within the *l*th tree (*t*). The first random effect accounts for the correlation between the observations taken on trees within the same status, while the second random effect accounts for the correlated observations within the single tree × height × zone. These random components account for the effects of pseudoreplication in our data set that is caused by sampling of the same trees at multiple heights and zones. The modeling was performed with ASReml-R (Butler et al. 2017) using the Kenward and Roger (2009) approximation to estimate degrees of freedom for the denominator, while the model-based pairwise comparisons were performed using asremlPlus (Brien 2020).

## Results

### Pathogen identification and anatomical observations

All the three diseased trees showed very advanced fibrous white rot in the heartwood at stump level, and part of the heartwood was easily disintegrating when cutting the thin stem sections for sampling. At stump level, the average sapwood width, reaction zone width, and decay column diameter were for the three sampled trees, respectively: 3.1, 1.5, and 28.4 cm for tree #1; 2.9, 1.8, and 25.0 cm for tree #2; 3.9, 2.4, and 16.9 cm for tree #3. At all the sampling heights, sapwood was free of decay around the stem circumference, indicating that the main route of stem infection was through the roots and not via any stem wound.

On fresh transverse discs from diseased trees, the reaction zone appeared as a characteristic yellow–brown–greenish discoloration ring (Fig. 1a). It is formed at the interface between sapwood and *Heterobasidion* colonized heartwood as a compartmentalization structure of the disease. This typical host–defense response was observed on discs at all the sampled stem heights of the diseased trees but absent in discs from healthy trees. At the interface between colonized heartwood and the reaction zone of diseased trees, an aniline-to-brown-colored zone was present, which here is referred to as discolored wood. In the reaction zone and discolored wood, tracheids, ray parenchyma and epithelial cells of traumatic resin ducts were often filled with dark brownish oxidized contents (Fig. 1b). Stem wood colonization covered the entire heartwood in all the discs sampled from the diseased trees, as conidiophores characteristic to *Heterobasidion* species were present from pith to the edge of the discolored wood bordering the reaction zone (Fig. 1c). Real-time PCR assays confirmed that the fungal DNA extracted from colonized wood belonged to *H. parviporum*, and not to *H. annosum* in any of the trees.

The diseased trees also revealed numerous tangentially aligned traumatic resin canals exuding resin drops, observed when the sampled sections were left in dark at room temperature for 7 days (Fig. 1c). Such belts of resin ducts (Fig. 1d), occurring within the early wood of specific year rings and mostly in association with the reaction zone, were not detected in healthy trees, which only contained single resin ducts randomly scattered in the sapwood. The reaction zone revealed marked accumulation of polyphenolics with specific auto-fluorescence, particularly in ray cells and adjacent tracheids (Fig. 1e). The traumatic resin canals formed an interconnected network with nearby ducts and rays and were lined by epithelial cells (Fig. 1f).

### Lignan profiles in wood

A total of 17 lignans were identified (Table 2 and Table S1). These were, in ascending order of retention time: 7-R-todolactol, isolariciresinol, liovil type I, secoisolariciresinol, todolactol isomer, α-conindendrin acid, 7-OH-lariciresinol, 9-OH-lariciresinol I, liovil type II, 7-hydroxymatairesinol isomer I, 9-OH-lariciresinol II, matairesinol, 7-hydroxymatairesinol isomer II, lariciresinol, α-conindendrin isomer, α-conindendrin, and pinoresinol. The reaction zone of the diseased trees showed the highest number of identified lignans, attaining on average 12.3, 11, and 9.3 lignans at 0, 1.6, and 3.2 m stem height, respectively (data not shown). Also noteworthy was the number of lignans in the heartwood of diseased and healthy trees, 2 and 8.5, respectively.

**Table 2:** Distribution of identified lignan and terpene compounds and their main classes in the different tissue zones of diseased and healthy trees, in ascending order of retention time (tR)

Compounds	Abbrev	tR (min)	DISEASED				HEALTHY		
			HW	DW	RZ	SW	HW		SW
*Lignans*									
7-R-Todolactol^c,f^	Todo	39.36	–	2.3	35	0.76	3.2		0.22
Isolariciresinol^g^	Iso	39.76	0.52	0.90	1.1	–	1.2		–
Liovil type I^d^	LioI	39.90	0.10	11	15	0.36	1.2		0.42
Secoisolariciresinol^a^	Sec	40.22	0.64	19	57	0.44	6.4		–
Todolactol isomer^e^	Todi	40.46	–	23	170	11	13		6.6
α-Conindendrin acid^b^	ConA	40.68	–	113	132	–	–		–
7-OH-Lariciresinol^c^	Lar7	41.09	0.10	11	45	1.1	1.8		0.27
9'-OH-Lariciresinol I^a^	Lar9I	41.58	–	–	5.8	0.26	0.24		0.15
Liovil type II^d^	LioII	41.61	–	8.9	29	0.61	2.1		0.74
Hydroxymatairesinol isomer I^a^	HMRI	41.90	7.5	201	541	54	62		52
9'-OH-Lariciresinol II^a^	Lar9II	41.91	–	6.3	53	3.0	2.1		–
Matairesinol^a^	Mat	41.93	0.01	66	207	1.5	0.58		–
Hydroxymatairesinol isomer II^a^	HMRII	42.06	0.16	124	527	5.7	–		0.10
Lariciresinol^a^	Lar	42.51	–	1.2	2.4	0.19	13		1.4
α-Conindendrin isomer^a^	Coni	42.87	3.3	297	202	0.4	4.4		0.16
α-Conindendrin^a^	Con	43.14	–	0.35	1.2	–	–		–
Pinoresinol^a^	Pino	44.10	0.53	6.2	6.3	12	2.3		2.4
Σ Total lignans			13	890	2030	91	114		64
*Terpenes*									
α-Pinene	aPin	4.16	5.3	1.4	2.9	3.9		1.9	9.4
β-Pinene	bPin	5.54	0.41	0.27	0.63	1.1		0.71	9.9
Sabinene	Sab	5.78	0.10	0.17	0.18	0.2		–	–
β-Elemene	Ele	14.27	0.17	–	0.76	0.6		–	–
α-Terpineol	Ter	16.50	0.32	0.53	0.19	–		–	–
Germacrene-D	Ger	16.64	–	0.22	–	8.5		–	0.90
σ-Cadiene	Cad	17.42	0.87	1.7	1.2	5.5		0.41	0.40
β-Springene	Spr	22.02	–	–	0.63	4.2		1.7	1.1
Thunbergene	Thbe	22.17	26	25	12	77		20	31
Verticillol	Vert	22.36	18	17	9.5	55		10	24
Neocembrene A	Neo	22.60	–	1.3	0.68	5.4		0.23	1.3
Trachylobane	Trac	23.01	–	2.2	–	6.6		–	2.0
Manoyloxide I	ManI	23.25	8.6	4.0	5.4	6.1		2.7	4.1
Manoyloxide II	ManII	23.26	14	6.6	9.0	12		6.9	8.3
Thunbergol	Thbo	24.25	33	38	26	118		58	81
Stachene	Stac	25.49	2.0	2.9	0.17	24		14	30
Σ Total terpenes			109	101	69	328		118	203

Mean concentration estimates (µg g^−1^ DW equivalent to nonadecanoic acid ISTD for lignans, and to pentadecane ISTD for terpenes) are based on samples taken from *n*  =  3 diseased trees at 0 m, and 1.6 m and 3.2 m stem height, and from *n*  =  3 healthy control trees at base (0 m) and 3.2 m stem height

*HW*  heartwood; *DW* discolored wood; *RZ* reaction zone; S*W* sapwood; *tR* retention time for lignans in HP-5 and terpenes in DB-WAx columns; *ISTD*  internal standard. Compounds not detected are delineated with a hyphen, -

Compound assignment verified by comparisons as follows: ^a^ Reference standard; ^b^ MS from literature (Ekman 1976); ^c^ MS and TLC data from literature (Willför et al. 2006); ^d^ Liovil type with dominant fragment at *m*/*z* 297, and missing *m*/*z* 484 and 223 (Willför et al. 2005; Smeds et al. 2012); ^e, f^ Todolactol isomer with dominant *m*/*z* 297 and low *m*/*z* 484, and 7R-todolactol with dominant *m*/*z* 297, low *m*/*z* 484 and *m*/*z* 323 dominating over *m*/*z* 324 (Smeds et al. 2012); ^g^ MS from literature (Yamamoto et al. 2004)

**Fig. 2:**
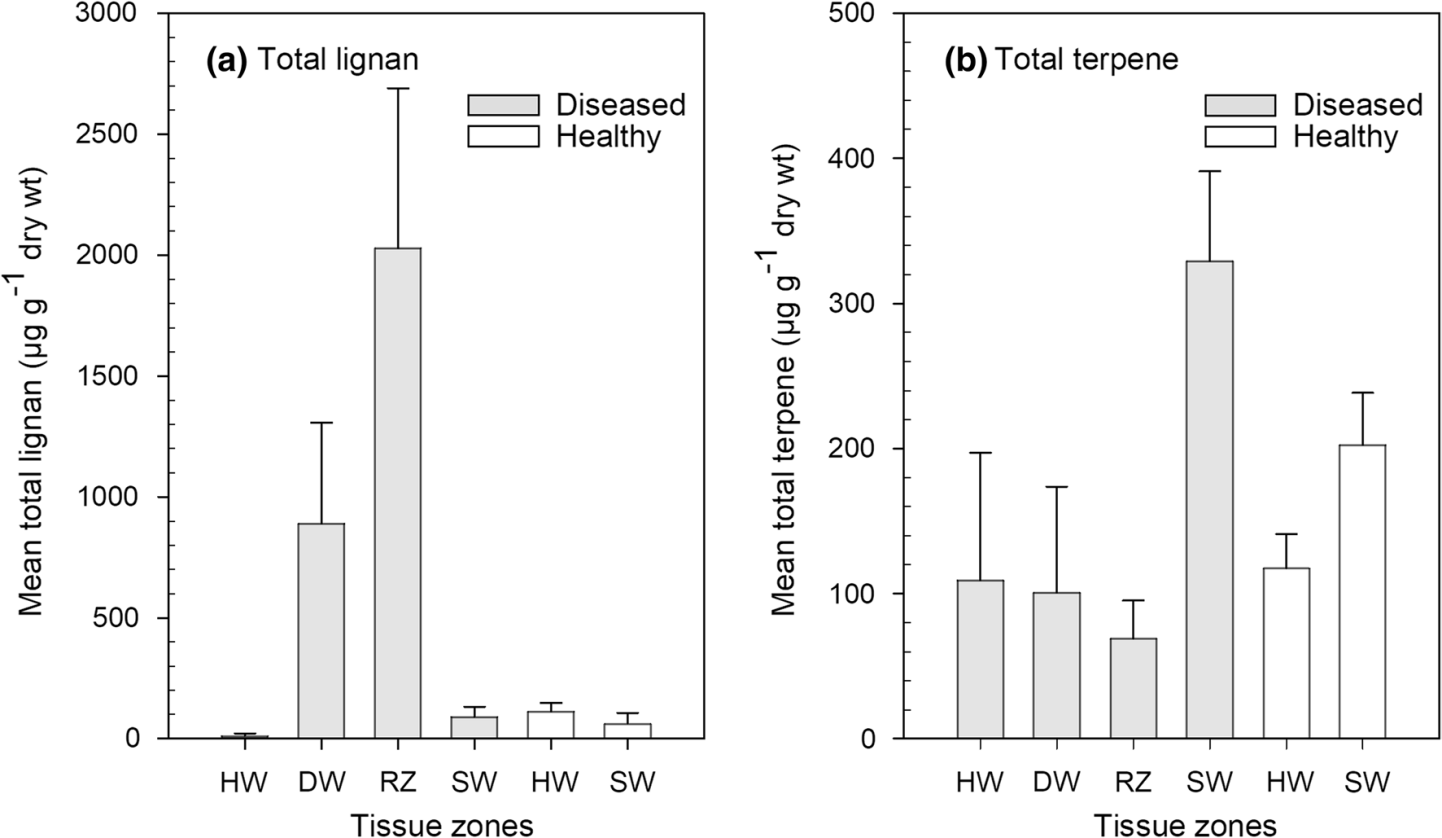
Concentration (mean ± SE, *n* = 3) of total lignan **a** and total terpene **b** in the four main tissue zones. Order of tissue zones from left to right: heartwood (HW), discolored wood (DW), reaction zone (RZ), and sapwood (SW) of diseased trees, and HW and SW of healthy trees

**Fig. 3:**
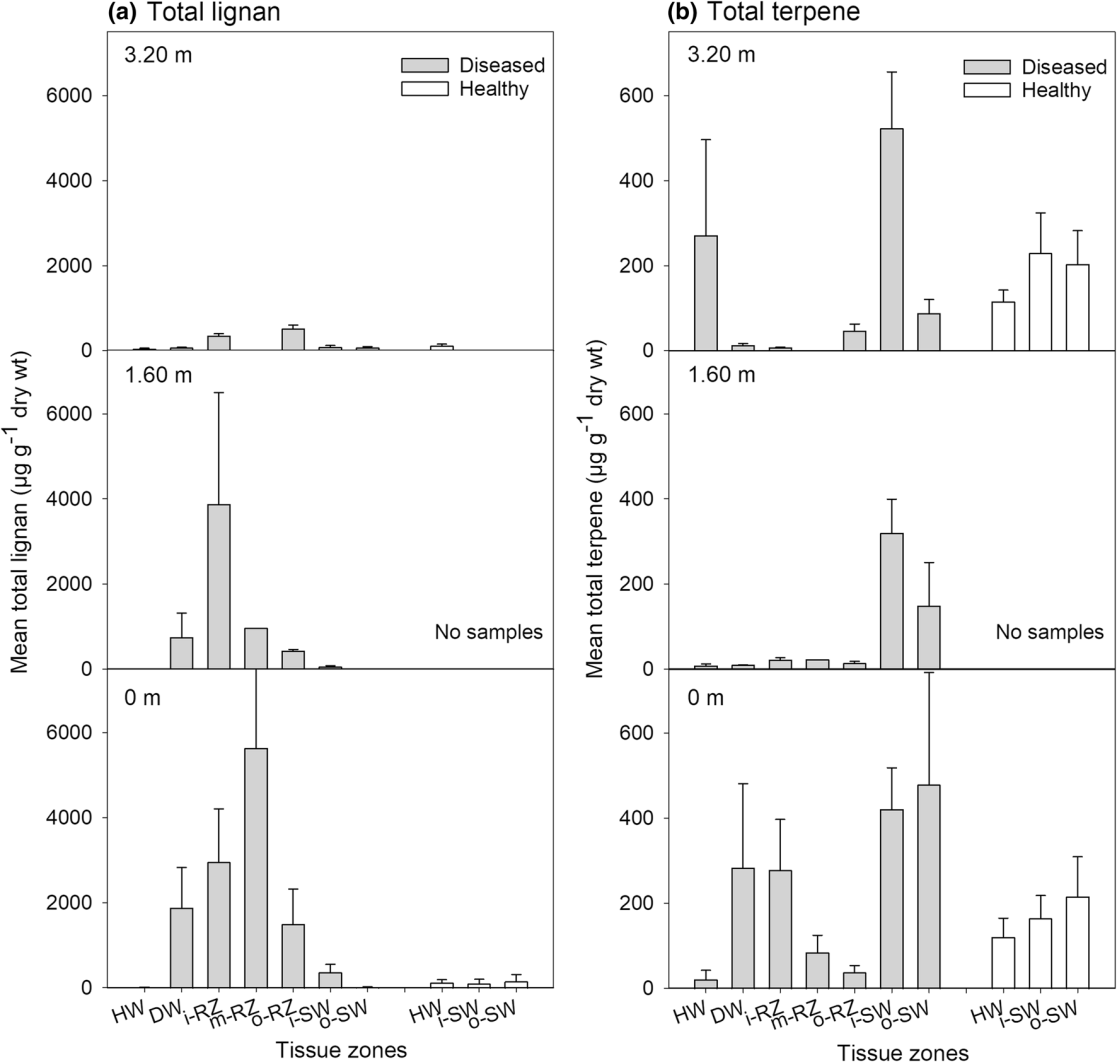
Concentration (mean ± SE, *n* = 3) of total lignan **a** and total terpene **b** in the four main tissue zones and the subzones at three different stem heights 0, 1.6, and 3.2 m. Order of tissue zones from left to right: heartwood (HW), discolored wood (DW), inner, middle and outer reaction zone (i, m, o-RZ), and inner (i-SW) and outer sapwood (o-SW) of diseased trees, and HW and inner and outer SW of healthy trees

The most lignan-rich tissues were the reaction zone and discolored wood of diseased trees, with a total lignan content of 2030 and 890 µg g^−1^, respectively (Fig. 2a, Table 2). The total lignan content within the heartwood and sapwood zones of diseased and healthy trees ranged from 13 to 114 µg g^−1^. The total concentration of lignans in the reaction zone and the discolored wood was significantly higher at 0 and 1.6 m in comparison to sapwood and heartwood within diseased trees and healthy trees (Fig. 3a, *P* < 0.05). There were much less-pronounced, non-significant differences in total lignan concentration between sampling heights of healthy trees (Fig. 3a, Table S1 and S2). Sapwood of healthy and diseased trees showed a low and comparable total lignan content, whereas the healthy trees revealed a significantly higher lignan content in heartwood in comparison to heartwood from diseased trees (*P* < 0.05).

**Fig. 4:**
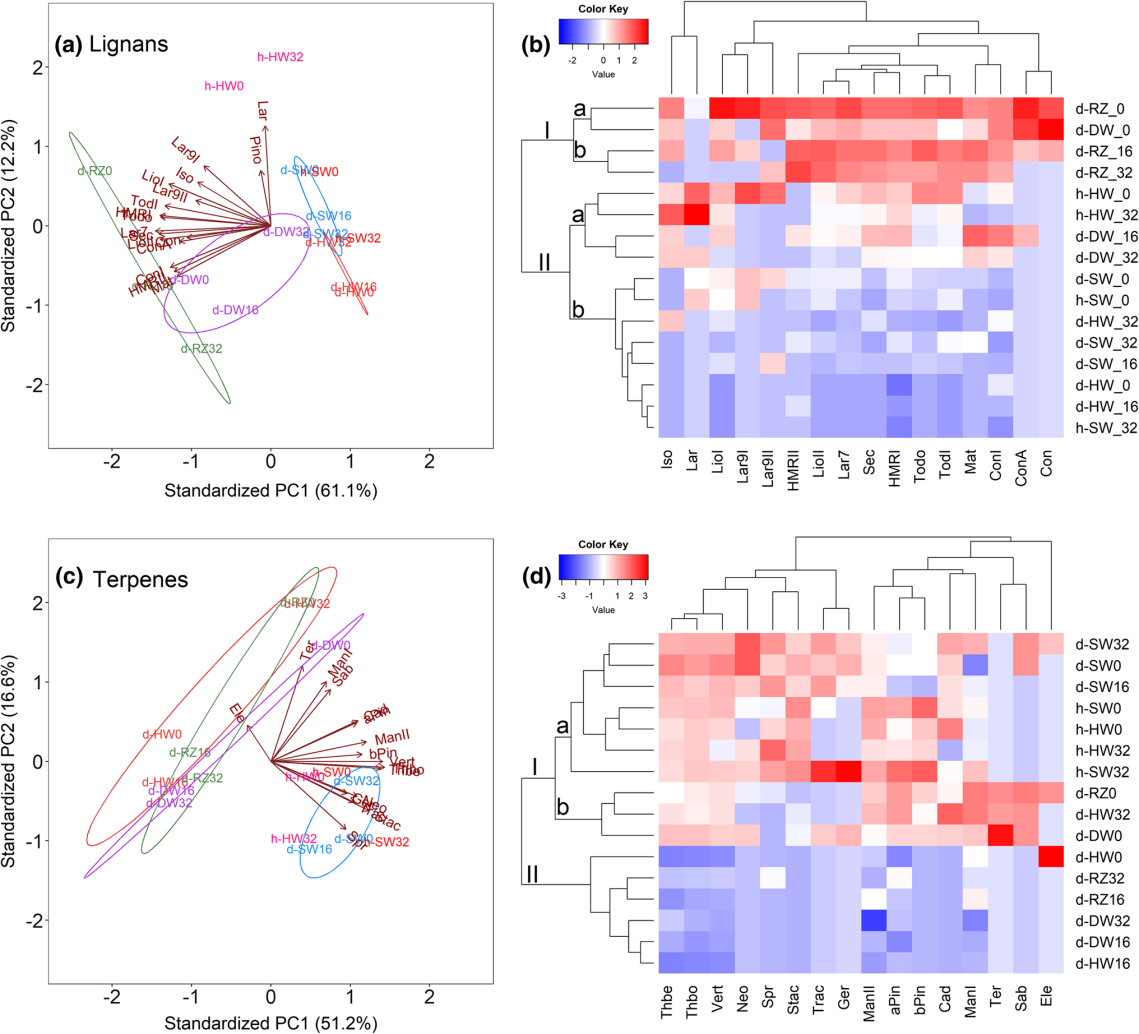
PCA biplots and heatmaps based on 17 lignan and 16 terpene compounds in wood zones and at different heights (0, 1.6, and 3.2 m) along the stem of diseased and healthy trees. **a, c** PCA biplots of the lignan and terpene compounds. Lignan and terpene loadings are symbolized by arrows (see also Table S3 for the loading values). The zone scores are depicted by their acronyms. **b, d** Heatmaps of the lignan and terpene compounds. The blue–red color indicates the deviation of the compound concentration from the mean (zero). Red indicates a higher content of the compound and blue a lower. Abbreviations: *d* diseased; *h*  healthy; *RZ* reaction zone; *DW* discolored wood; *SW* sapwood; *HW* heartwood

Principal component analysis (PCA) provided an overview of the lignan and terpene data sets, with tissue-type clustering based on characteristic compounds. For lignans, the first two PCA axes represented 61.1% and 12.2% of the total variance (Fig. 4a). The lignans formed several groups of highly correlated variables, most of which correlated with PCA1 and showed positive association with samples from the reaction zone and discolored wood (excluding d-DW at 3.2 m height), but were negatively associated with samples from sapwood of healthy and diseased trees (Fig. 4a). The basal lignans in the lignan biosynthesis pathway (pinoresinol and lariciresinol), correlated with PCA2 and were positively associated with heartwood samples of healthy trees (Table S3), showed no or negative association with the reaction zone and discolored wood samples. In comparison to PCA, the high-dimensional heatmap combined with hierarchical clustering showed essentially a similar pattern, with some differences in the sub-clustering of discolored wood samples from 1.6 and 3.2 m heights and in the correlation of individual lignans (Fig. 4b).

The most pronounced result from the ANOVA was the highly significant tissue zone effects shown by 13 lignans (Table S2). A health status effect was observed for four of these compounds: matairesinol, 7-hydroxymatairesinol isomer II, lariciresinol, and α-conindendrin isomer. Reaction zone and the discolored wood of the diseased trees had significantly higher concentrations than healthy heartwood of nine and six lignans, respectively (*P* < 0.05). In contrast, healthy heartwood had significantly higher concentrations of four lignans (todolactol isomer, lariciresinol, 9-OH-lariciresinol, and 7-hydroxymatairesinol isomer I) than diseased heartwood (*P* < 0.05). Lignans that were prominent in the reaction zone and the discolored wood of the diseased trees, but absent or detected only at a negligible level in healthy trees included matairesinol, 7-hydroxymatairesinol isomer II, α-conindendrin acid, and α-conidendrin isomer.

### Terpene profiles in wood

A total of 16 terpenes were identified from wood samples (Table 2 and Table S4): three monoterpenes (α-pinene, β-pinene, sabinene), three sesquiterpenes (β-elemene, germacrene D, σ-cadiene), seven diterpenes (β-springene, thunbergene, neocembrene A, trachylobane, manoyloxide I, manoyloxide II, stachene), two monoterpene alcohols (α-terpineol, verticillol), and one diterpene alcohol (thunbergol). Diterpenes and diterpene alcohols formed on average ca. 40% and 50%, respectively, of the total terpene content in the different tissue types of healthy and diseased trees, the rest being primarily represented by monoterpenes and sesquiterpenes. The number of identified terpenes was quite stable across tissues and sampling heights in healthy trees, with average values of 9.5 and 8.2 specific terpenes detected in sapwood and heartwood, respectively (derived from Table S4). In diseased trees, on average, 7.8, 4.5, 4.7, and 3.1 specific terpenes were detected in sapwood, reaction zone, discolored wood, and heartwood, respectively. The lowest terpene diversity was detected in heartwood at stump level, where on average 1.0 specific terpenes occurred.

The averaged total terpene concentration across all sampling heights in the heartwood, discolored wood, reaction zone, and sapwood of diseased trees (Fig. 2b, Table 2) was 109, 101 69, and 328 µg g^−1^, respectively, with the latter differing significantly from the other tissues (*P* < 0.05). For healthy trees, the terpene content in heartwood and sapwood was 118 and 203 µg g^−1^, respectively (Fig. 2b, Table 2). In the sapwood of healthy trees, the highest terpene content was recorded at 3.2 m height, whereas in the diseased trees, the highest amount occurred at stump level (Fig. 3b).

For terpenes, the first two PCA axes represented 51.2% and 16.6% of the total variance (Fig. 4c). Mono-, sesqui-, and diterpenes formed several groups of highly correlated variables, most of which correlated with PCA1 and showed positive association with samples from sapwood of diseased and healthy trees and healthy heartwood, but negative association with the reaction zone and discolored wood samples from 1.6 and 3.2 m heights, and diseased heartwood from 0 and 1.6 m heights. α-Terpineol, manoyloxide I, and sabinene correlated best with PCA2 and showed positive association with samples from the reaction zone and discolored wood at 0 m height and from diseased heartwood at 3.2 m height. The heatmap combined with hierarchical clustering showed essentially a similar sub-grouping of tissue types as the PCA. Diterpenes showed generally higher correlation to each other than mono- and sesquiterpenes (Fig. 4d).

The ANOVA showed a significant tissue zone effect for seven terpenes, while the stem height effect proved significant for five terpenes (Table S5). The effect of health status was significant only for β-pinene and stachene, sapwood of healthy trees showing significantly higher concentration of these compounds than sapwood of diseased trees. In the healthy trees, sapwood showed significantly higher concentrations of α-pinene, β-pinene, trachylobane, stachene, and germacrene D than heartwood (*P* < 0.05). In the diseased trees, sapwood showed significantly higher concentrations of β-springene, thunbergene, neocembrene A, trachylobane, stachene, thunbergol, and verticillol than the reaction zone, discolored wood and heartwood.

The terpene profile from pure resin in droplets exuded from axial traumatic resin canals and pockets of diseased sapwood differed considerably from the terpene profile of extracted wood of diseased and healthy trees (Table S6). Above 80% of terpenes associated with the axial traumatic resin canals and the resin pocket were monoterpenes (predominantly α-pinene, β-pinene, and verbenone) and monoterpene alcohols (predominantly cis-carveol), the rest sesquiterpenes, diterpenes, and diterpene alcohols.

### Wood mass loss induced by Fenton reaction

**Fig. 5:**
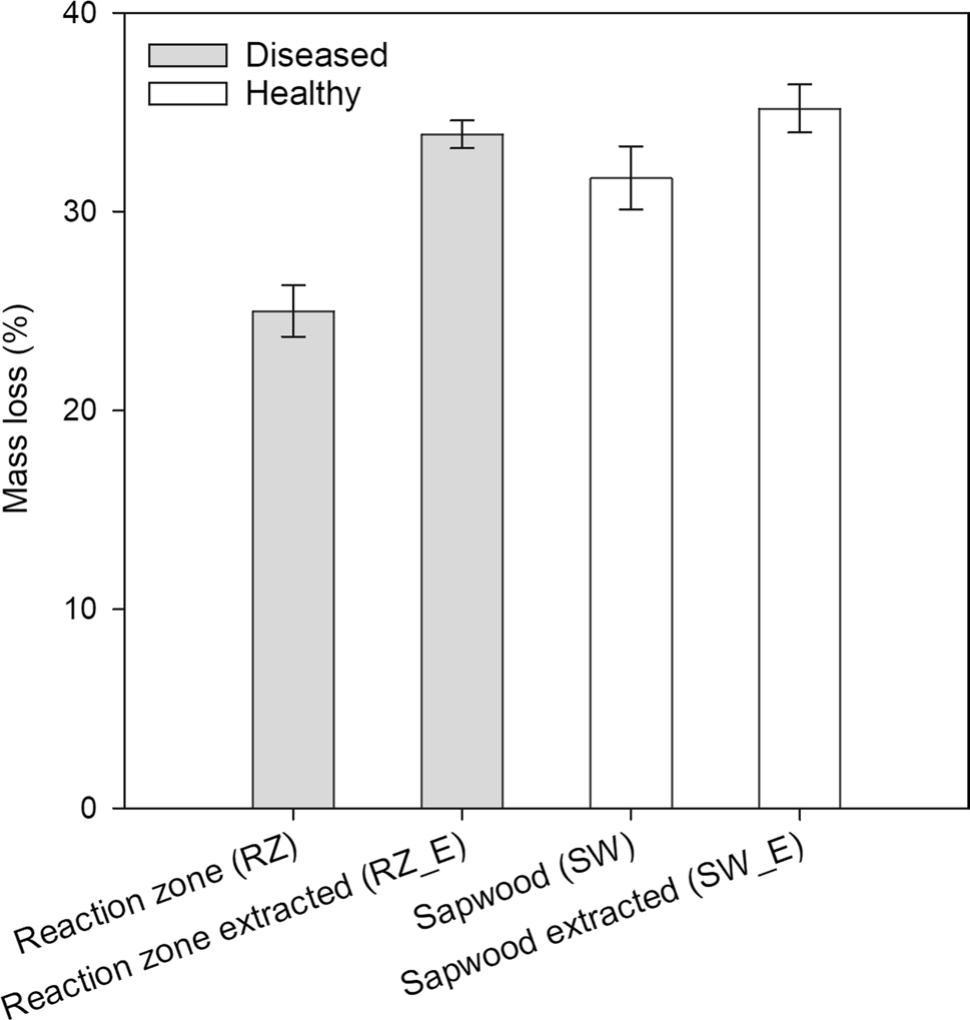
Mass loss of Norway spruce sapwood and reaction zone when subjected to Fenton treatment. Mass loss was measured for unextracted and acetone extracted (E) wood powders from the reaction zone (RZ) tissues of diseased trees and the inner sapwood (SW) of healthy trees (mean ± SE, *n* = 5 samples in each group)

To consider the putative role of phenolic extractives in wood resistance to oxidative degradation, we subjected unextracted and acetone extracted powder from reaction zone and sapwood to Fenton treatment, used as a proxy for oxidative decomposition of wood by decay fungi, and recorded afterward wood mass loss. The 25% mass loss recorded for unextracted reaction zone was significantly lower (Tukey HSD, *P* < 0.01) than the 32–35% mass losses recorded for extracted reaction zone and unextracted and extracted healthy sapwood (Fig. 5).

## Discussion

Tree xylem is composed primarily of dead tracheary elements hosting a large amount of structural carbohydrates in their cell wall. The living xylem parenchyma—associated with radially orientated rays and radially and axially orientated resin ducts in conifers—forms a three-dimensional network that is considered instrumental in defense-related signaling, biosynthesis, and transport against xylem pathogens (Nagy et al. 2000; Morris et al. 2020). In the current study, the diseased trees showed intact sapwood around stem circumference, indicating that stem heartwood decay originated from root infection. Since colonization of Norway spruce heartwood by *Heterobasidion* species progresses upwards at a rate of 30–40 cm per year (Hallaksela 1993; Bendz-Hellgren et al. 1999), our analysis of samples from stump level and 1.6 and 3.2 m stem heights provides an approximately 8–10-year-long window into tree–pathogen interaction history. Here we profiled the amount of two major defense-related secondary metabolite groups, lignans and terpenes, in Norway spruce stems naturally infected by the heartwood-based white-rot fungus *Heterobasidion parviporum*.

### Lignan profiles and tree–pathogen interaction

To our knowledge, the present study is the first one to document lignan profiles at different stem heights in *Heterobasidion*-infected trees. The trees affected by heartwood decay had formed a so-called reaction zone at the interface between colonized wood and sapwood to compartmentalize the infection. This defense zone involves local accumulation of lignans and metal ions, leading to elevation of wood pH close to 7, while that of healthy wood is around 5 (Shain 1971; Johansson and Theander 1974). The total concentration of lignans was three-to-tenfold higher in the reaction zone at stump height and at 1.6 m level than at 3.2 m height (Fig. 3a). While the highest levels of lignans were recorded in the reaction zone margin facing colonized wood, a relatively high lignan content was also observed at the reaction zone margin adjacent to sapwood at stump level.

It can be envisaged that strengthening of the reaction zone by lignan deposition takes place during several years to achieve a deep impregnation of the tissue. The spatial variation in the occurrence of lignans within the reaction zone is in line with histological observations that phenol-occluded tracheids can form continuous tangential belts in some regions of this defense xylem, whereas in other areas, only the ray cells are filled with phenols (Johansson and Theander 1974; Hietala et al. 2009).

The host responses to heartwood decay can be captured by comparison of lignan profiles of intact reaction zone and healthy heartwood. Lignans are considered to be synthesized mainly in ray cells at the narrow sapwood–heartwood transition zone (Davin and Lewis 2000; Suzuki and Umezawa 2007). In consistency, the highest diversity and concentration of lignans in healthy and diseased trees was observed in the tissues aligning the transition zone, i.e., heartwood in healthy trees and reaction zone in diseased trees. It is noteworthy that the lignans hydroxymatairesinol isomer II, matairesinol, and α-conindendrin acid were present in the reaction zone of all the diseased trees but generally absent in healthy trees. This could be caused by pathway differences in lignan synthesis between the healthy and diseased trees, but it may also be due to the specific matrix properties of the reaction zone. Under alkaline conditions, hydroxymatairesinol may form α-conidendric acid and hydroxymatairesinol isomer II or matairesinol (Eklund et al. 2004). Besides high H_2_O content, the reaction zone contains oxalate (Nagy et al. 2012a) and metals such as cobalt (Hietala et al. 2016), conditions that may facilitate the transformation of lignan compounds.

The fate of lignans in pathogen-colonized wood can be elucidated by comparison of lignan profiles of intact reaction zone and heartwood with those of respective colonized tissues. Previously, we proposed that the fungal colonized discolored heartwood, adjacent to reaction zone, represents a compromised reaction zone, because it shows a pH of ca. 6, which is intermediate between that of reaction zone (pH around 7) and normal heartwood (pH 5) (Nagy et al. 2012a,b). The elevated lignan concentrations now observed at stump level in the discolored wood of diseased trees in comparison to healthy heartwood support this scenario. Feeding of white-rot fungi on lignocellulose in the reaction zone requires pH modulation as the pH optimum of their lignin-degrading manganese peroxidases is typically below 5 (Kuan et al. 1993). *H. parviporum* secretes oxalic acid to lower local pH via chelation of the excessive cations present in the reaction zone (Nagy et al. 2012a). Under acidic conditions, such as in case when treated with formic acid, isomers of hydroxymatairesinol and α-conidendric acid can be converted to α-conidendrin (Freudenberg and Knof 1957; Goldschmid and Hergert 1961).

It seems plausible that the observed trend of increase in the relative proportion of α-conidendrin isomer in the infected trees across reaction zone—discolored wood—heartwood is partly due to wood acidification-related conversion of hydroxymatairesinol isomers, the two most prominent lignans in the reaction zone. The observation that both the total amount and diversity of lignans were higher in heartwood of healthy trees in comparison to that of diseased trees (Fig. 2) also indicates that *H. parviporum* is able to decompose lignans. The α-conidendrin can serve as the sole carbon source for microorganisms—bacterial use of α-conidendrin has been reported to proceed as follows: α-conidendrin → vanillic acid → *p*-hydroxybenzoic acid → protocatechuic acid → ketoadipic acid (Tabak et al. 1959). We are not aware of any similar studies in relation to wood decay fungi. Laboratory experiments with lignans as the sole carbon source for *H. parviporum* are needed to clarify this.

Our experimental data showed that removal of phenolic extractives from the reaction zone significantly increased wood mass loss by Fenton reaction generated hydroxyl free radicals (Fig. 5). Thus, the antioxidant properties of lignans may interfere with the oxidative processes employed by wood decay fungi to break down lignin, which necessitate their removal, so that these fungi can access the structural cell wall carbohydrates. Enzymes potentially involved in lignan decomposition by *Heterobasidion* species include laccases (Popoff et al. 1975; Yakovlev et al. 2013). Concerning the question of whether individual lignans could be also directly toxic to *Heterobasidion* species, the findings of prior studies remain somewhat inconclusive. Shain and Hillis (1971) reported that matairesinol and hydroxymatairesinol slowed down slightly the growth rate of a *Heterobasidion* isolate on agar conditions at the test concentrations between 0.1% and 0.4%, whereas conidendrin had no inhibitory effect. Of the nine lignans and the catechol 4-methylcatechol identified from the reaction zone of Norway spruce by Popoff et al. (1975), the catechol was the most potent growth inhibitor of *Heterobasidion*, followed by the lignan liovil, whereas even the maximum test concentration of hydroxymatairesinol (2% in agar) had no effect on the growth of *Heterobasidion*. Taken together, these studies would indicate that the predominant lignans of Norway spruce, hydroxymatairesinol, matairesinol, and α-conindendrin, are not very toxic to *Heterobasidion*, but that some of the lignans may have, either alone or in combination with other phenols, also growth inhibitory effects.

Histological observations indicate that *H. parviporum* hyphae, as is typical for white-rot fungi, are pressed against the innermost secondary cell wall layer (S3) upon xylem delignification (Hietala et al. 2009). While the cellular localization of lignans in the reaction zone is unclear, polyphenolic substances, besides filling ray parenchyma, fill also the lumina of early wood and latewood tracheids or coat the S3 wall in this tissue (Hietala et al. 2009). It would indicate that these substances are synthesized and/or transported in ray parenchyma and are present in close enough proximity to fungal attack on the tracheid cell wall to have any effect.

The mass losses observed for unextracted reaction zone were comparable to those observed for heartwood and knotwood of Scots pine upon subjection to Fenton reaction (Belt et al. 2017). Scots pine is an extractive-rich tree species with generally higher wood decay resistance than Norway spruce. It should be noted that, unlike brown-rot fungi, white-rot fungi do not employ Fenton reaction but diffusible low molecular redox mediators, such as Mn^3+^ (Janusz et al. 2017). However, lignans can be expected to scavenge also white-rot-associated redox mediators, as hydroxymatairesinol, matairesinol, lariciresinol, and secoisolariciresinol, derived from Norway spruce knotwood, have been demonstrated to scavenge superoxide radicals and peroxyl radicals (Willför et al. 2003; Pietarinen et al. 2006). Broken branches are common infection routes for wood decay fungi, and it appears likely that the accumulation of lignans in the reaction zone and knotwood serve a similar purpose, protection of sapwood against these pathogens.

### Terpene profiles and heartwood compartmentalization

In contrast to phenylpropanoids, terpene-based defenses are regarded as some of the most expensive secondary compounds for plants to synthesize (Gershenzon 1994). In the Italian Alps, Vezzola et al. (2019) analyzed the mono- and sesquiterpene contents of the last 5-year rings in the sapwood of healthy Norway spruce trees, and trees with natural heartwood infection by *H. parviporum*. They recorded slightly higher total concentration of monoterpenes in healthy trees in comparison to diseased ones, whereas the relative proportions of the monoterpenes α-pinene and β-pinene were significantly greater in infected trees in comparison to healthy trees. Also in the present study, the total monoterpene content was somewhat higher in the sapwood of healthy trees in comparison to the diseased trees, whereas the sapwood from uninfected trees had significantly higher α-pinene and β-pinene content than sapwood from diseased trees. The reason for this difference between the two studies is not clear. Apart from the paper by Vezzola et al. (2019) and the current one, we are not aware of any other study that has investigated the level of terpenes in Norway spruce showing natural heartwood infection by *Heterobasidion* species.

One noteworthy result of the current study was the visual observation of the presence of traumatic resin canals deep in the stem xylem of infected trees. It has been demonstrated that both mock inoculation and wounding-assisted artificial stem bark inoculation of Norway spruce with *Heterobasidion* trigger the formation of traumatic resin canals in the newly forming year ring (Krekling et al. 2004). It is quite possible the traumatic resin canals now observed in the diseased trees represent an induced defense response triggered already when the pathogen was still confined to roots as these canals must have formed during the year, in which the corresponding year rings were formed. Further studies are needed to determine whether the localization of traumatic ducts within specific year rings of stem wood could be used to approximate the age of initial root infection by *Heterobasidion* species. We are not aware of any prior observations of traumatic resin canals in stems with natural heartwood colonization by *Heterobasidion*.

While diterpenes and diterpene alcohols formed above 90% of the terpenes detected in bulk sapwood samples from healthy and diseased trees, the resin from traumatic resin canals formed in the diseased trees was predominated by monoterpenes (especially α-pinene, β-pinene, and verbenone) and monoterpene alcohols (especially cis-carveol), which constituted above 80% of total terpene content. Monoterpene-rich resin is very fluid, a property that could facilitate resin flow into cavities formed by *Heterobasidion* in the reaction zone, both to seal these damages to prevent ingress of air into sapwood and to suppress further lateral spread of the fungus.

In simplified laboratory experiments, resin acids (abietic and dehydroabietic acids) and several monoterpenes have been shown to inhibit growth of *Heterobasidion* species in a dose-dependent manner. Of the tested compounds, the monoterpene (+)-α-pinene and oxygenated monoterpenes α-terpineol and bornyl acetate were most effective in slowing down the growth of *H. parviporum* (Henriks et al. 1979; Kusumoto et al. 2014). *Heterobasidion* species are obviously able to hydroxylate dehydroabietic acid (Ekman and Sjöholm 1979), and thus potentially detoxify them. A study published by Gibbs (1968) shows that resin yields of pines and the ability to mobilize resin were correlated with resistance to *Heterobasidion annosum* (syn. *F. annosus*). Finally, the epithelial cells of traumatic resin ducts have been shown to contain phenylalanine ammonia lyase (EC 4.3.1.5), indicating synthesis of phenolics as a possible resin component (Nagy et al. 2000).

The role of terpenoids as part of the induced defenses against heartwood-based stem colonization by *Heterobasidion,* both from a specific and a system biology perspective, remains inconclusive. The organization of traumatic resin ducts as tangentially aligned belts is a unique orientation in comparison to the radially aligned ray parenchyma. Considering this organization and the constitutively formed parenchyma and resin channels in the xylem, further research is warranted about the role of traumatic resin ducts in heartwood compartmentalization related signal transduction, synthesis, and translocation.

## Summary and conclusions

The study is based on analyses of polyphenols of the class lignans, and terpene resins in fungus-attacked and healthy Norway spruce trees at three stem heights (0, 1.6, and 3.2 m) and in four tissue types (sapwood, reaction zone, discolored wood, and heartwood). The wood tissues showed marked differences in the amount and diversity of lignans and terpenes—while lignans were associated with the reaction zone of compromised wood, with decreasing trend with stem height reflecting upwards reducing propagation of infection, terpenes showed maximum levels in the sapwood but less-pronounced upward reduction.

The comparison of chemical profiles between healthy and diseased trees and between intact and pathogen-compromised wood, coupled with wood mass loss determination upon Fenton test, indicates that antioxidant properties of lignans hinder processes utilized by wood decay fungi for lignocellulose decomposition. However, the fungus is eventually able to decompose lignans, α-conindendrin isomer possibly serving as an intermediate metabolite in lignan decomposition.

The diseased trees showed numerous tangentially aligned traumatic resin canals in the reaction zone, which probably represent an induced defense response triggered when the pathogen was still confined to roots. The resins of the canals contained primarily monoterpenes, while the sapwood of healthy trees contained mainly diterpenes. Compartmentalization effects against oxidative degradation by fungi are revealed by lignan impregnation of the reaction zone in *Heterobasidion*-infected wood. Considering that the traumatic resin canals contain alive cells that connect reaction zone tissues at different heights and at different stages of host–pathogen interaction, further research is warranted whether these organs may play roles in signal transduction, synthesis, and translocation of defense compounds in relation to formation and strengthening of the reaction zone.

## *Author contribution statement*

AMH and NEN conceived and designed the research and conducted the experiments. HRN and MF performed the chemical analyses; IMH the molecular analyses and RBØ microscopy. JD developed statistical models and analyzed the data. AMH, HRN, JD, and NEN prepared the original draft. All authors revised and approved the manuscript.

## Supplementary Information

Below is the link to the electronic supplementary material.

Supplementary file1 (DOCX 104 KB)

## Data Availability

The authors declare that all data supporting the findings of this study are available within the article and its supplementary information files. The raw datasets generated from chemical analyses during the current study are available from the corresponding author (NEN) on request.
