# Supplementary material for: Patterns and roles of lignan and terpenoid accumulation in the reaction zone compartmentalizing pathogen-infected heartwood of Norway spruce
Source: Planta. 2022 Feb 10;255(3):63. doi: 10.1007/s00425-022-03842-1 (PMC8831285; doi:10.1007/s00425-022-03842-1)
Supplement: Supplementary file 1 — Supplementary file1 (DOCX 104 KB) [file 425_2022_3842_MOESM1_ESM.docx]

**Patterns and roles of lignan and terpenoid accumulation in the reaction zone compartmentalizing pathogen-infected heartwood of Norway spruce**

**Nina Elisabeth Nagy^1^, Hans Ragnar Norli^1^, Monica Fongen^1^, Runa Berg Østby^3^, Inger M. Heldal^1^, Jahn Davik^1^, Ari M. Hietala^2^**

^1^Norwegian Institute of Bioeconomy Research, P.B. 115, NO-1431 Ås, Norway

^2^Norwegian Institute of Bioeconomy Research, P.B. 2609, NO-7734 Steinkjer, Norway

^3^Østfold University College, Faculty of Health, Welfare and Organisation, P.B. 700, NO-1757 Halden, Norway

Corresponding authors: [nina.nagy@nibio.no](mailto:nina.nagy@nibio.no), Orcid-ID 0000-0001-8371-4751; [ari.hietala@nibio.no](mailto:ari.hietala@nibio.no), Orcid-ID 0000-0002-3195-6318

| **Table S1** Raw data of the identified lignan compounds in diseased and healthy trees at all heights and each tissue zone. Mean concentration estimates (µg g^-1^ DW equivalent to nonadecanoic acid, ISTD) are based on samples taken from *n* = 3 trees in each group at stem base (0 m), 1.6 m and 3.2 m stem height, *n* = 2 samples from each tissue zone, and outer (o), inner (i) and mid part (m) of the sapwood and reaction zone tissues, and similarly tissues from control trees at stem base (0 m) and 3.2 m stem height. Abbreviations: HW = heartwood; DW = discoloured wood; RZ = reaction zone; SW= sapwood; *t*R = retention time for lignans in a HP-5 column; ISTD = internal standard. Compounds not detected are delineated with a hyphen, - | | | | | | | | | | | | |
| --- | --- | --- | --- | --- | --- | --- | --- | --- | --- | --- | --- | --- |
| Lignans | *t*R (min)  HP-5 | Height (m) | Diseased | | | | | | | Healthy | | |
|  |  |  | HW | DW | i-RZ | m-RZ | o-RZ | i-SW | o-SW | HW | i-SW | o-SW |
| 7-R-Todolactol _c,f_ | 39.36 | 0 | - | 3.6 | 55 | 171 | 21 | 2.6 | - | 5.4 | 0.88 | - |
|  |  | 1.6 | - | 0.74 | 25 | 14 | 7.4 | 1.7 | - |  |  |  |
|  |  | 3.2 | - | 2.6 | 3.7 |  | 6.0 | - | 0.31 | 0.99 | - | - |
| Isolariciresinol _g_ | 39.76 | 0 | - | 1.8 | 2.0 | 1.3 | 0.35 | - | - | 0.28 | - | - |
|  |  | 1.6 | - | 0.36 | 4.2 | - | 0.20 | - | - |  |  |  |
|  |  | 3.2 | 1.4 | 0.53 | - |  | - | - | - | 2.2 | - | - |
| Liovil type I _d_ | 39.90 | 0 | - | 19 | 27 | 24 | 11 | 1.2 | 0.36 | 0.91 | 0.21 | 1.5 |
|  |  | 1.6 | - | 13 | 46 | - | 0.88 | 0.24 | 0.20 |  |  |  |
|  |  | 3.2 | 0.25 | 0.19 | 0.49 |  | - | 0.17 | - | 1.4 | - | - |
| Secoisolariciresinol _a_ | 40.22 | 0 | - | 34 | 104 | 124 | 41 | 1.1 | - | 4.6 | - | - |
|  |  | 1.6 | - | 18 | 111 | 29 | 14 | 1.3 | - |  |  |  |
|  |  | 3.2 | 1.7 | 3.3 | 8.5 |  | 15 | - | - | 8.3 | - | - |
| Todolactol isomer _e_ | 40.46 | 0 | - | 54 | 333 | 666 | 165 | 47 | - | 22 | 4.7 | 22 |
|  |  | 1.6 | - | 12 | 135 | 29 | 20 | 9.3 | - |  |  |  |
|  |  | 3.2 | - | 4.1 | 9.5 |  | 18 | 1.9 | 5.1 | 4.6 | - | - |
| α-Conindendrin acid _b_ | 40.68 | 0 | - | 317 | 141 | 736 | 6.3 | - | - | - | - | - |
|  |  | 1.6 | - | 22 | 243 | - | - | - | - |  |  |  |
|  |  | 3.2 | - | - | - |  | - | - | - | - | - | - |
| 7-OH-Lariciresinol (Lignan A) _c_ | 41.09 | 0 | - | 25 | 70 | 170 | 29 | 4.4 | - | 2.2 | 1.1 | - |
|  |  | 1.6 | - | 8.0 | 66 | 9.7 | 7.5 | 1.3 | - |  |  |  |
|  |  | 3.2 | 0.18 | 0.56 | 5.1 |  | 7.8 | - | 1.0 | 1.5 | - | - |
| 9'-OH-Lariciresinol I | 41.58 | 0 | - | - | 12 | 21 | 9.4 | 1.6 | - | 0.47 | - | 0.61 |
|  |  | 1.6 | - | - | 3.8 | - | - | - | - |  |  |  |
|  |  | 3.2 | - | - | - |  | - | - | - | - | - | - |
| Liovil type II _d_ | 41.61 | 0 | - | 9.4 | 38 | 33 | 12 | 1.7 | 1.1 | 2.0 | 2.0 | 0.95 |
|  |  | 1.6 | - | 17 | 86 | 25 | 9.2 | 0.19 | - |  |  |  |
|  |  | 3.2 | - | 0.49 | 8.1 |  | 14 | 0.56 | 0.11 | 2.1 | - | - |
| 7-Hydroxymatairesinol isomer I _a_ | 41.90 | 0 | 0.23 | 371 | 681 | 1602 | 401 | 250 | 0.89 | 67 | 85 | 120 |
|  |  | 1.6 | 1.3 | 207 | 1041 | 344 | 92 | 22 | 0.64 |  |  |  |
|  |  | 3.2 | 19 | 25 | 120 |  | 129 | 4.7 | 47 | 57 | 0.82 | 0.49 |
| 9'-OH-Lariciresinol II | 41.91 | 0 | - | 19 | 45 | 372 | 54 | 15 | - | 4.2 | - | - |
|  |  | 1.6 | - | - | - | - | - | 2.8 | - |  |  |  |
|  |  | 3.2 | - | - | - |  | 3.6 | - | - | - | - | - |
| Matairesinol _a_ | 41.93 | 0 | 0.04 | 34 | 196 | 294 | 210 | 1.9 | - | 1.2 | - | - |
|  |  | 1.6 | - | 154 | 502 | 236 | 72 | - | - |  |  |  |
|  |  | 3.2 | - | 8.4 | 61 |  | 85 | 2.7 | 4.6 | - | - | - |
| 7-Hydroxymatairesinol isomer II _a_ | 42.06 | 0 | - | 199 | 916 | 894 | 449 | 32 | - | - | - | 0.34 |
|  |  | 1.6 | 0.42 | 172 | 1045 | 187 | 177 | - | - |  |  |  |
|  |  | 3.2 | - | - | 102 |  | 196 | 1.4 | 0.38 | - | - | - |
| Lariciresinol _a_ | 42.51 | 0 | - | - | - | - | 16 | 1.2 | - | 8.9 | 2.4 | 3.2 |
|  |  | 1.6 | - | - | - | - | - | - | - |  |  |  |
|  |  | 3.2 | - | 3.5 | - |  | - | - | - | 16 | - | - |
| α-Conindendrin isomer _a_ | 42.87 | 0 | 5.1 | 779 | 313 | 516 | 68 | 1.0 | 0.21 | 7.3 | 0.40 | 0.23 |
|  |  | 1.6 | 0.73 | 107 | 543 | 79 | 14 | 0.30 | 0.62 |  |  |  |
|  |  | 3.2 | 4.7 | 4.9 | 17 |  | 27 | - | 0.26 | 1.4 | - | - |
| α-Conindendrin _a_ | 43.14 | 0 | - | 1.1 | 1.1 | 5.9 | - | - | - | - | - | - |
|  |  | 1.6 | - | - | 3.0 | - | - | - | - |  |  |  |
|  |  | 3.2 | - | - | - |  | - | - | - | - | - | - |
| Pinoresinol _a_ | 44.10 | 0 | - | 15 | 20 | 11 | 4.9 | 2.3 | 11 | 0.32 | 2.0 | 2.4 |
|  |  | 1.6 | - | 2.7 | 8.4 | - | 0.28 | 3.7 | 2.1 |  |  |  |
|  |  | 3.2 | 1.4 | 0.60 | - |  | 0.63 | 56 | 2.3 | 4.3 | 1.9 | 3.4 |
| \| Compound assignments verified by comparison as follows: ^a^ reference standard; ^b^ MS from literature (Ekman 1976); ^c^ MS and TLC data from literature (Willför et al. 2006); ^d^ Liovil type with dominant fragment at m/z 297, and missing m/z 484 and 223 (Willför et al. 2005; Smeds et al. 2012); ^e, f^ Todolactol isomer with dominant m/z 297 and low m/z 484, and 7R-todolactol with dominant m/z 297, low m/z 484 and m/z 323 dominating over m/z 324 (Smeds et al. 2012); ^g^ MS from literature (Yamamoto et al. 2004) \| \| --- \| | | | | | | | | | | | | |

| **Table S2** Significance levels from mixed model ANOVA of log transformed lignan quantities from healthy and diseased Norway spruce trees | | | | |
| --- | --- | --- | --- | --- |
|  | Fixed main effects | | | Significant random variance components^1^ |
| Compounds | Status (S) | Height (H) | Zone (Z)^2^ | Tree# x H x Z |
| 7-R-Todolactol |  |  | *** |  |
| Isolariciresinol |  |  |  |  |
| Liovil type I |  |  | ** |  |
| Secoiisolariciresinol |  |  | *** |  |
| Todolactol isomer |  |  | *** |  |
| α-Conindendrin acid | . |  | ** |  |
| 7-OH-Lariciresinol | . |  | *** |  |
| 9'-OH-Lariciresinol I |  |  |  |  |
| Liovil type II |  |  | ** |  |
| 7-Hydroxymatairesinol I |  |  | *** |  |
| 9'-OH-Lariciresinol II |  |  |  |  |
| Matairesinol | ** |  | *** | * |
| Hydroxymatairesinol II | * |  | *** |  |
| Lariciresinol | ** |  | *** |  |
| α-Conindendrin isomer | ** |  | *** |  |
| α-Conindendrin |  |  |  |  |
| Pinoresinol |  |  | * |  |
| Total lignan content | . |  | *** |  |
| Signif. codes: *** *P* < 0.001; ** *P* < 0.01; * *P* < 0.05; . *P* < 0.1  ^1^ Random effects are included in order to account for the possible correlated observations. The statistical model included several levels of such random factors. However, only at the experimental unit level (tree number x height x sampling zone) were such correlations observed, and hence that is the only random component reported in the table. ^2^ Zones, encompassing sapwood (SW), reaction zone (RZ), and discoloured wood (DW), heartwood (HW), are nested within status in the statistical modelling | | | | |

| **Table S3** Loadings of lignans and terpenes on the five first principal component (PC) axes derived from the R function ‘prcomp’. Loadings indicate how well the variable is represented by the principal component. *t*R = retention times for lignans in HP-5 and terpenes in DB-WAx columns | | | | | | | | |
| --- | --- | --- | --- | --- | --- | --- | --- | --- |
|  | Abbreviations | *t*R (min) | PC1 | PC2 | PC3 | PC4 | PC5 |  |
| **LIGNANS** |  |  |  |  |  |  |  |  |
| 7-R-Todolactol | Todo | 39.36 | -0.29 | 0.05 | -0.12 | 0.18 | -0.08 |  |
| Isolariciresinol | Iso | 39.76 | -0.19 | 0.26 | -0.14 | -0.59 | -0.13 |  |
| Liovil type I | LioI | 39.90 | -0.27 | 0.25 | 0.14 | -0.07 | -0.01 |  |
| Secoisolariciresinol | Sec | 40.22 | -0.30 | -0.05 | -0.20 | -0.08 | -0.04 |  |
| Todolactol isomer | Todi | 40.46 | -0.28 | 0.12 | -0.20 | 0.21 | 0.12 |  |
| α-Conindendrin acid | ConA | 40.68 | -0.24 | -0.09 | 0.42 | -0.26 | -0.14 |  |
| 7-OH-Lariciresinol | Lar7 | 41.09 | -0.30 | -0.03 | -0.02 | 0.09 | 0.06 |  |
| 9'-OH-Lariciresinol I | Lar9I | 41.58 | -0.18 | 0.36 | 0.10 | 0.43 | -0.16 |  |
| Liovil type II | LioII | 41.61 | -0.29 | -0.08 | -0.09 | 0.12 | 0.17 |  |
| Hydroxymatairesinol isomer I | HMRI | 41.90 | -0.29 | 0.06 | -0.15 | -0.06 | 0.18 |  |
| 9'-OH-Lariciresinol II | Lar9II | 41.91 | -0.20 | 0.15 | 0.31 | 0.35 | -0.33 |  |
| Matairesinol | Mat | 41.93 | -0.25 | -0.30 | -0.16 | -0.10 | 0.22 |  |
| Hydroxymatairesinol isomer II | HMRII | 42.06 | -0.26 | -0.27 | -0.03 | 0.18 | 0.27 |  |
| Lariciresinol | Lar | 42.51 | -0.02 | 0.59 | -0.35 | -0.15 | -0.08 |  |
| α-Conindendrin isomer | Coni | 42.87 | -0.27 | -0.25 | -0.09 | -0.22 | -0.22 |  |
| α-Conindendrin | Con | 43.14 | -0.22 | -0.07 | 0.46 | -0.20 | -0.21 |  |
| Pinoresinol | Pino | 44.10 | -0.03 | 0.33 | 0.42 | -0.12 | 0.73 |  |
|  |  |  |  |  |  |  |  |  |
| **TERPENES** |  |  |  |  |  |  |  |  |
| α-Pinene _a_ | aPin | 4.16 | 0.26 | 0.20 | 0.34 | 0.14 | 0.21 |  |
| β-Pinene _a_ | bPin | 5.54 | 0.27 | 0.04 | 0.37 | 0.19 | 0.09 |  |
| Sabinene _a_ | Sab | 5.78 | 0.18 | 0.38 | -0.42 | -0.17 | -0.02 |  |
| β-Elemene _b_ | Ele | 14.27 | -0.07 | 0.19 | -0.26 | 0.50 | -0.63 |  |
| α-Terpineol _d_ | Ter | 16.50 | 0.09 | 0.50 | -0.14 | -0.06 | 0.32 |  |
| Germacrene D _b_ | Ger | 16.64 | 0.23 | -0.17 | -0.21 | 0.44 | 0.41 |  |
| σ-Cadiene _b_ | Cad | 17.42 | 0.26 | 0.21 | 0.01 | -0.27 | -0.13 |  |
| β-Springene _c_ | Spr | 22.02 | 0.22 | -0.35 | 0.02 | 0.04 | -0.29 |  |
| Thunbergene _c_ | Thbe | 22.17 | 0.33 | -0.04 | -0.09 | -0.27 | -0.05 |  |
| Verticillol _d_ | Vert | 22.36 | 0.33 | 0.00 | -0.08 | -0.18 | -0.06 |  |
| Neocembrene A _c_ | Neo | 22.60 | 0.26 | -0.18 | -0.45 | -0.03 | -0.06 |  |
| Trachylobane _c_ | Trac | 23.01 | 0.25 | -0.21 | -0.25 | 0.36 | 0.27 |  |
| Manoyloxide I _c_ | ManI | 23.25 | 0.16 | 0.42 | 0.11 | 0.36 | -0.13 |  |
| Manoyloxide II _c_ | ManII | 23.26 | 0.28 | 0.10 | 0.30 | 0.09 | -0.23 |  |
| Thunbergol _b_ | Thbo | 24.25 | 0.34 | -0.03 | -0.02 | -0.18 | -0.09 |  |
| Stachene _c_ | Stac | 25.49 | 0.29 | -0.26 | 0.21 | -0.04 | -0.13 |  |
|  |  |  |  |  |  |  |  |  |
| Compound assignments: ^a^ monoterpene, ^b^ sesquiterpene, ^c^ diterpene, ^d^ terpenoid alcohols | | | | | | | | |

| **Table S4** Raw data of the identified terpene compounds in diseased and healthy trees at all heights and each tissue zone. Mean concentration estimates (µg g^-1^ DW equivalent to nonadecanoic acid, ISTD) values are based on samples taken from *n* = 3 trees in each group at stem base (0 m), 1.6 m and 3.2 m stem height, *n* = 2 samples from each tissue zone, and outer (o), inner (i) and mid part (m) of the sapwood and reaction zone tissues, and similarly tissues from control trees at stem base (0 m) and 3.2 m stem height. Abbreviations: HW = heartwood; DW = discolored wood; RZ = reaction zone; SW= sapwood; *t*R = retention time for terpenes in a DB-WAx column; ISTD = internal standard. Compounds not detected are delineated with a hyphen, - | | | | | | | | | | | | | | |
| --- | --- | --- | --- | --- | --- | --- | --- | --- | --- | --- | --- | --- | --- | --- |
| Terpenes | *t*R (min) | Height (m) | Diseased | | | | | | |  | | Healthy | | |
|  |  |  | HW | DW | i-RZ | m-RZ | o-RZ | i-SW | o-SW |  | | HW | i-SW | o-SW |
| α-Pinene _a_ | 4.16 | 0 | - | 3.6 | 5.7 | 4.2 | 6.8 | 13 | 6.0 |  | 1.3 | | 4.7 | 4.0 |
|  |  | 1.6 | 0.48 | - | 0.53 | - | 0.6 | 1.2 | - |  |  | |  |  |
|  |  | 3.2 | 14 | 0.58 | 1.6 |  | 1.6 | 1.8 | 1.3 |  | 2.5 | | 14 | 15 |
| β-Pinene _a_ | 5.54 | 0 | - | 0.80 | 1.1 | 1.2 | 2.3 | 3.9 | 1.5 |  | 0.62 | | 4.6 | 4.6 |
|  |  | 1.6 | - | - | - | - | - | - | - |  |  | |  |  |
|  |  | 3.2 | 1.1 | - | - |  | - | 0.97 | 0.30 |  | 0.79 | | 12 | 18 |
| Sabinene _a_ | 5.78 | 0 | - | 0.51 | 1.1 | - | 0.16 | 0.2 | 0.10 |  | - | | - | - |
|  |  | 1.6 | - | - | - | - | - | - | - |  |  | |  |  |
|  |  | 3.2 | 0.13 | - | - |  | - | 0.60 | 0.3 |  | - | | - | - |
| β-Elemene _b_ | 14.27 | 0 | 0.7 | - | 4.2 | 1.3 | - | - | - |  | - | | - | - |
|  |  | 1.6 | - | - | - | - | - | - | - |  |  | |  |  |
|  |  | 3.2 | - | - | - |  | - | 3.6 | - |  | - | | - | - |
| α-Terpineol _d_ | 16.50 | 0 | - | 1.6 | 1.1 | 0.20 | - | - | - |  | - | | - | - |
|  |  | 1.6 | - | - | - | - | - | - | - |  |  | |  |  |
|  |  | 3.2 | 0.90 | - | - |  | - | - | - |  | - | | - | - |
| Germacrene D _b_ | 16.64 | 0 | - | 0.70 | - | - | - | 11 | 15 |  | - | | - | - |
|  |  | 1.6 | - | - | - | - | - | 5.6 | - |  |  | |  |  |
|  |  | 3.2 | - | - | - |  | - | 19 | 0.20 |  | - | | 2.4 | 1.2 |
| σ-Cadiene _b_ | 17.42 | 0 | - | 5.2 | 5.9 | 3.5 | - | 2.9 | 4.8 |  | 0.80 | | - | 1.1 |
|  |  | 1.6 | - | - | - | - | - | 2.2 | 0.40 |  |  | |  |  |
|  |  | 3.2 | 2.3 | - | - |  | - | 23 | - |  | - | | 0.50 | - |
| β-Springene _c_ | 22.02 | 0 | - | - | 3.5 | - | - | 5.9 | 6.0 |  | 1.5 | | 1.6 | - |
|  |  | 1.6 | - | - | - | - | - | 5.2 | 3.1 |  |  | |  |  |
|  |  | 3.2 | - | - | - |  | 0.78 | 5.2 | - |  | 1.8 | | 1.9 | 0.88 |
| Thunbergene _c_ | 22.17 | 0 | - | 68 | 55 | 16 | 3.1 | 99 | 140 |  | 19 | | 33 | 34 |
|  |  | 1.6 | - | 1.4 | 2.4 | - | - | 82 | 38 |  |  | |  |  |
|  |  | 3.2 | 70 | 4.7 | - |  | 8.2 | 90 | 16 |  | 21 | | 31 | 25 |
| Verticillol _d_ | 22.36 | 0 | - | 51 | 44 | 12 | 2.9 | 72 | 97 |  | 15 | | 22 | 27 |
|  |  | 1.6 | - | 0.40 | 2.1 | 1.1 | - | 56 | 27 |  |  | |  |  |
|  |  | 3.2 | 49 | 0.80 | - |  | 6.5 | 68 | 13 |  | 6.0 | | 26 | 22 |
| Neocembrene A _c_ | 22.60 | 0 | - | 3.9 | 4.5 | - | - | 5.6 | 11 |  | - | | - | 2.0 |
|  |  | 1.6 | - | - | - | - | - | 3.3 | 1.9 |  |  | |  |  |
|  |  | 3.2 | - | - | - |  | - | 9.9 | 1.1 |  | 0.54 | | 1.2 | 1.9 |
| Trachylobane _c_ | 23.01 | 0 | - | 6.5 | - | - | - | 6.1 | 17 |  | - | | - | 0.76 |
|  |  | 1.6 | - | - | - | - | - | 4. 8 | 3.0 |  |  | |  |  |
|  |  | 3.2 | - | - | - |  | - | 8.7 | - |  | - | | 5.4 | 2.1 |
| Manoyloxide I _c_ | 23.25 | 0 | 7.2 | 9.3 | 14 | 6.5 | 5.1 | 4.1 | 2.4 |  | 2.7 | | 3.8 | 4.3 |
|  |  | 1.6 | 2.1 | 1.5 | 3.5 | 6.7 | 3.6 | 9.2 | 3.1 |  |  | |  |  |
|  |  | 3.2 | 16 | 1.2 | 2.0 |  | 2.1 | 13 | 5.2 |  | 2.8 | | 2.8 | 5.3 |
| Manoyloxide II _c_ | 23.26 | 0 | 13 | 14 | 18 | 12 | 10 | 11 | 13 |  | 7.9 | | 7.8 | 9.7 |
|  |  | 1.6 | 4.2 | 4.2 | 6.7 | 11 | 7.7 | 15 | 9.2 |  |  | |  |  |
|  |  | 3.2 | 24 | 1.6 | 2.9 |  | 3.6 | 17 | 8.4 |  | 5.8 | | 8.3 | 7.3 |
| Thunbergol _b_ | 24.25 | 0 | - | 110 | 120 | 27 | 7.0 | 159 | 132 |  | 59 | | 61 | 96 |
|  |  | 1.6 | - | 0.71 | 5.4 | 2.1 | 0.64 | 114 | 50 |  |  | |  |  |
|  |  | 3.2 | 88 | 2.2 | - |  | 23 | 216 | 39 |  | 58 | | 90 | 75 |
| Stachene _c_ | 25.49 | 0 | - | 8.7 | - | 1.7 | - | 28 | 349 |  | 14 | | 26 | 31 |
|  |  | 1.6 | - | - | - | - | - | 20 | 13 |  |  | |  |  |
|  |  | 3.2 | 5.2 | - | - |  | - | 47 | 2.5 |  | 153 | | 33 | 29 |
|  |  |  |  |  |  |  |  |  |  |  |  | |  |  |
| **Σ Monoterpenes _a_** |  |  | **15** | **5.5** | **10** | **5.4** | **12** | **22** | **9.5** |  | **5.1** | | **36** | **41** |
| **Σ Sesquiterpenes _b_** |  |  | **3.0** | **5.8** | **10** | **4.7** | **0** | **67** | **21** |  | **0.8** | | **2.9** | **2.3** |
| **Σ Diterpenes _c_** |  |  | **142** | **125** | **112** | **54** | **44** | **489** | **327** |  | **92** | | **156** | **154** |
| **Σ Terpenoid alcohol_d_** |  |  | **138** | **167** | **173** | **42** | **40** | **685** | **3576** |  | **137** | | **199** | **220** |
| Compound assignments: ^a^ monoterpene; ^b^ sesquiterpene; ^c^ diterpene; ^d^ terpenoid alcohols | | | | | | | | | | | | | | |
|  | | | | | | | | | | | | | | |

| **Table S5** Significance levels from mixed model ANOVA of log transformed terpen quantities from healthy and diseased Norway spruce trees | | | | |
| --- | --- | --- | --- | --- |
|  | Fixed main effects | | | Significant random variance components^1^ |
| Compounds | Status (S) | Height (H) | Zone (Z)^2^ | Tree# x H x Z |
| α-Pinene _a_ |  | * |  | ** |
| β-Pinene _a_ | ** |  | * | ** |
| Sabinene _a_ |  |  |  | * |
| β-Elemene _b_ |  |  |  |  |
| α-Terpineol |  | * |  |  |
| Germacrene D _b_ |  |  | ** | . |
| σ-Cadiene _b_ |  |  |  |  |
| β-Springene _c_ |  |  |  |  |
| Thunbergene _c_ |  | * | ** |  |
| Verticillol _d_ |  | ** | ** |  |
| Neocembrene A _c_ |  |  | ** |  |
| Trachylobane _c_ |  |  | . |  |
| Manoyloxide I _c_ |  |  |  | * |
| Manoyloxide II _c_ |  |  |  | * |
| Thunbergol _b_ | . | ** | ** |  |
| Stachene _c_ | *** |  | *** |  |
| Monoterpenes |  | * |  | ** |
| Sesquiterpenes |  |  |  | . |
| Diterpenes |  |  | *** |  |
| Terpenoid alcohols |  | ** | ** |  |
| Total terpenoid content | * | * | *** |  |
| Compound assignments: ^a^ monoterpene, ^b^ sesquiterpene, ^c^ diterpene, ^d^ terpenoid alcohols  Signif. codes: *** *P* < 0.001; ** *P* < 0.01; * *P* < 0.05; . *P* < 0.1  ^1^ Random effects are included in order to account for the possible correlated observations. The statistical model included several levels of such random factors. However, only at the experimental unit level (tree number x height x sampling zone) were such correlations observed, and hence that is the only random component reported in the table. ^2^ Zones, encompassing sapwood (SW), reaction zone (RZ), and discoloured wood (DW), heartwood (HW), are nested within status in the statistical modelling | | | | |

| **Table S6** Terpenes extracted from resin droplets exuded from reaction zone associated traumatic resin ducts at 0 m height and from one resin pocket at 3.2 m height. Concentrations are given in µg g^-1^ pentadecane equivalents. RZ = Reaction zone; *t*R = retention times for lignans in HP-5 and terpenes in DB-WAx columns | | | | | | | | | |
| --- | --- | --- | --- | --- | --- | --- | --- | --- | --- |
|  | | |  |  | Tree1,  0m RZ | Tree 2,  0m RZ | Tree 3,  0m RZ |  | Tree 2,  3.2m RZ |
| Compounds | *t*R (min) | | | | Resin droplets from traumatic ducts | | |  | Resin pocket |
|  | | |  |  |  |  |  |  |  |
| α-Pinene _a_ | | 4.27 |  | 2900 | | 1100 | 2500 |  | 2200 |
| Camphene _a_ | | 4.91 |  | - | | - | 700 |  | 1100 |
| β-Pinene _a_ | | 5.56 |  | 550 | | 700 | 3100 |  | 14000 |
| Sabinene _a_ | | 5.86 |  | - | | - | - |  | 210 |
| Limonene _a_ | | 7.29 |  | - | | - | 180 |  | 1000 |
| γ-Terpinene _a_ | | 8.23 |  | - | | - | - |  | 50 |
| *para*-Cymene _a_ | | 8.66 |  | 60 | | 20 | 1900 |  | 330 |
| α-Cubebene _b_ | | 12.33 |  | - | | - | - |  | 130 |
| α-Longipinene _b_ | | 12.48 |  | - | | - | - |  | 720 |
| Ylangene _b_ | | 12.79 |  | - | | - | - |  | 300 |
| Longicyclene _b_ | | 13.05 |  | - | | - | - |  | 130 |
| Sativene _b_ | | 13.56 |  | - | | - | - |  | 50 |
| α-Gurjunene _b_ | | 13.99 |  | - | | - | - |  | 1500 |
| β-Elemene _b_ | | 14.28 |  | - | | - | - |  | 1700 |
| α-Cedrene _b_ | | 14.29 |  | - | | 440 | 1000 |  | - |
| Terpinene-4-ol _d1_ | | 14.92 |  | - | | - |  |  | 0.04 |
| *cis*-Carveol _d1_ | | 16.23 |  | 3500 | | 1000 | 12000 |  | 1700 |
| γ-Muurolene _b_ | | 16.33 |  | - | | - | - |  | 3500 |
| α-Terpineol _d1_ | | 16.52 |  | - | | - | - |  | 40 |
| Borneol _a_ | | 16.58 |  | - | | - | - |  | 50 |
| Verbenone _a_ | | 16.65 |  | 3500 | | 1000 | 12000 |  | 530 |
| Valencene _b_ | | 16.92 |  | - | | - | - |  | 110 |
| σ-Cadiene _c_ | | 17.47 |  | - | | - | - |  | 600 |
| Thunbergene _c_ | | 22.19 |  | - | | - | 1900 |  | 600 |
| Verticillol _d2_ | | 22.38 |  | - | | - | - |  | 400 |
| Manoyloxide _c_ | | 23.17 |  | 880 | | 210 | - |  | 890 |
| Thunbergol _d2_ | | 24.28 |  | - | | - | 2500 |  | 540 |
| Epimanool _d2_ | | 24.73 |  | - | | - | 3400 |  |  |
|  | | |  |  |  |  |  |  |  |
| Σ Monoterpenes _a_ | | |  |  | 6900 | 2900 | 43000 |  | 40000 |
| Σ Sesquiterpenes _b_ | | |  |  | - | 440 | 1400 |  | 5000 |
| Σ Diterpenes _c_ | |  |  | 880 | | 210 | 1900 |  | 2100 |
| Σ Monoterpenoid alcohols _d1_ | |  |  | 3500 | | 9600 | 12000 |  | 1800 |
| Σ Diterpenoid alcohols _d2_ | |  |  | - | | - | 5900 |  | 980 |
| Σ Total terpenoid alcohols _d_ | | |  |  | 3500 | 9600 | 1800 |  | 2600 |
| Σ Total terpenoid content _a,b,c,d_ | | |  |  | 11000 | 4400 | 65000 |  | 49000 |
| Compound assignments: ^a^ monoterpene, ^b^ sesquiterpene, ^c^ diterpene, ^d^ terpenoid alcohol, ^d1^ monoterpenoid alcohol, ^d2^ diterpenoid alcohol | | | | | | | | | |
